# Alder and the Golden Fleece: high diversity of *Frankia* and ectomycorrhizal fungi revealed from *Alnus glutinosa* subsp. *barbata* roots close to a Tertiary and glacial refugium

**DOI:** 10.7717/peerj.3479

**Published:** 2017-07-18

**Authors:** Melanie Roy, Adrien C. Pozzi, Raphaëlle Gareil, Melissande Nagati, Sophie Manzi, Imen Nouioui, Nino Sharikadze, Patricia Jargeat, Hervé Gryta, Pierre-Arthur Moreau, Maria P. Fernandez, Monique Gardes

**Affiliations:** 1 Laboratoire Evolution Diversité Biologique (EDB UMR 5174), Université Toulouse 3 Paul Sabatier, CNRS, ENFA, Toulouse, France; 2 Laboratoire Ecologie Microbienne (UMR5557), Université Claude Bernard (Lyon I), CNRS, Villeurbanne, France; 3 Department of Neurobiology , Ilia State University, Tbilisi, Georgia; 4 Laboratoire Impact de la Diversité Chimique sur la Santé Humaine (IMPECS, EA 4483), CHU, Institut Pasteur, Université du Droit et de la Sante (Lille II), Lille, France

**Keywords:** *Frankia*, Ectomycorrhiza, Colchis, *Alnus*, Alnicola, Glacial refugia, Tertiary refugia

## Abstract

**Background:**

Recent climatic history has strongly impacted plant populations, but little is known about its effect on microbes. Alders, which host few and specific symbionts, have high genetic diversity in glacial refugia. Here, we tested the prediction that communities of root symbionts survived in refugia with their host populations. We expected to detect endemic symbionts and a higher species richness in refugia as compared to recolonized areas.

**Methods:**

We sampled ectomycorrhizal (EM) root tips and the nitrogen-fixing actinomycete *Frankia* communities in eight sites colonized by *Alnus glutinosa* subsp*. barbata* close to the Caucasus in Georgia. Three sites were located in the Colchis, one major Eurasian climatic refugia for Arcto-Tertiary flora and alders, and five sites were located in the recolonized zone. Endemic symbionts and plant ITS variants were detected by comparing sequences to published data from Europe and another Tertiary refugium, the Hyrcanian forest. Species richness and community structure were compared between sites from refugia and recolonized areas for each symbionts.

**Results:**

For both symbionts, most MOTUs present in Georgia had been found previously elsewhere in Europe. Three endemic *Frankia* strains were detected in the Colchis *vs* two in the recolonized zone, and the five endemic EM fungi were detected only in the recolonized zone. *Frankia* species richness was higher in the Colchis while the contrary was observed for EM fungi. Moreover, the genetic diversity of one alder specialist *Alnicola xanthophylla* was particularly high in the recolonized zone. The EM communities occurring in the Colchis and the Hyrcanian forests shared closely related endemic species.

**Discussion:**

The Colchis did not have the highest alpha diversity and more endemic species, suggesting that our hypothesis based on alder biogeography may not apply to alder’s symbionts. Our study in the Caucasus brings new clues to understand symbioses biogeography and their survival in Tertiary and ice-age refugia, and reveals that isolated host populations could be of interest for symbiont diversity conservation.

## Introduction

Glacial refugia are recognized sources of genetic diversity and hot spots of taxonomic diversity (Médail & Diadema, 2009). They are also key regions for studying biogeography, as recently shown for fungi (Geml et al., 2010; Ghobad-Nejhad et al., 2012; Merényi et al., 2014). In Europe, the numerous fossil records from glacial periods and the analysis of allelic diversity have contributed to identification of refugia (Taberlet & Cheddadi, 2002; Gavin et al., 2014), mainly in Italy, the Iberian Peninsula and the Balkans (Médail & Diadema, 2009). Demographic contractions and expansions, and population genetic patterns associated with ice age refugia have been deeply studied in oaks, pines, beech, and alders (Taberlet & Cheddadi, 2002; Hampe & Jump, 2011). Interestingly, all these tree species host mutualistic fungi on their roots, and are ectomycorrhizal (EM; Smith & Read, 2008). However, whether these biogeographic and genetic processes play out similarly in microbes is unknown. In obligatory host-associated microbes, the potential for joint genetic responses to extinction and recolonization is high. On the other hand, they could have also survived outside of refugia, either in spore banks, or associated with other hosts. The existing knowledge about tree biogeography offers a framework to compare their symbiont communities inside and out of refugia, and test the hypothesis that symbionts have survived with their host in glacial refugia,

The recent history of *Alnus* has been intensely studied in Europe (King & Ferris, 1998; Douda et al., 2014; Havrdová et al., 2015; Mandák et al., 2016a; Mandák et al., 2016b). Compared to timber trees, alders are ideal models for biogeographical studies of historical migrations because there are numerous natural populations with low human impact (Douda et al., 2014). Thanks to molecular markers, southern refugia have been detected for *A. glutinosa* (L.) Gaertn. in the Iberian, Apennine, Corsica, North Africa, Balkan and Anatolian Peninsulas (King & Ferris, 1998). More recently, the existence of both refugia in Belarus and western Russia has been demonstrated; they were important sources for northward post-glacial expansion of alders (Douda et al., 2014). Today, *A. glutinosa* populations are still marked by recent isolation in glacial refugia (King & Ferris, 1998), and several unique chloroplast haplotypes have been detected in relict populations from Morocco (Lepais et al., 2013) and Turkey (King & Ferris, 1998; Havrdová et al., 2015). Of interest, the high genetic diversity detected in Turkey concurs with the observed morphological variations of *A. glutinosa* in this region. Therefore, four subspecies are recognized in this region: *A. glutinosa* subsp*. glutinosa, A. glutinosa* subsp*. antitaurica* Yalt, *A.  glutinosa* subsp*. betuloides* Ansin, and *A. glutinosa* subsp*. barbata* (CA Meyer) Yalt.

*Alnus glutinosa* subsp*. barbata* is distributed from Turkey to Iran, and is considered as a Tertiary relict plant, common in the Colchis forests of Georgia. Pollen records from the Quaternary era and distribution modeling have revealed that *Alnus* swamps were extended in Colchis floodplains and existed continuously over the last 10,000 years (Connor & Kvavadze, 2009). Today, stands of *A. glutinosa* subsp*. barbata* still dominate the Colchis floodplain (Nakhutsrishvili, 2012). Located in the South Caucasus, the Colchis region is one of the three major refugia of Tertiary relict taxa worldwide (Milne & Abbott, 2002; Kikvidze & Ohsawa, 2001; Denk, Frotzler & Davitashvili, 2001). Because a mountain ridge blocks rains coming from the Black Sea, the Colchis floodplain is well separated from arid lowlands and steppes from Eastern Georgia (Nakhutsrishvili, 2012). According to pollen records, alder recolonized Eastern Georgia and the central mountain ridge around 2,000 years BP, and are represented today by isolated populations (Connor & Kvavadze, 2009). Adjacent to the Colchis forests of Georgia, the other major Western Eurasia climate refugium for temperate forests is the Hyrcanian forest, located on the southern coast of the Caspian Sea in the region of Iran and southern Azerbaidjan. The two refugia lie approximately 2,000 km apart, and are now completely isolated from each other by steppe vegetation. However, they still share several Tertiary plant relict species, and are considered to be the two oldest temperate deciduous forests in western Eurasia (Maharramova et al., 2015).

As compared to other tree species, alders host few lineages of EM fungi (Molina, 1981; Moreau, Peintner & Gardes, 2006; Moreau et al., 2011; Rochet et al., 2011) and species-poor EM communities (Põlme et al., 2013; Roy et al., 2013; Kennedy, Walker & Bogar, 2015). Among frequently encountered EM taxa, *Alnicola* and *Alpova* are two genera that appear to be strictly associated with alders because they have never been found on any other tree species. Several species of *Lactarius, Russula, Amanita* and *Cortinarius* are also exclusive to alders. Alders also associate with nitrogen-fixing actinobacteria, all belonging to the genus *Frankia* (Weber, Nurmiaho-Lassila & Sundman, 1987) and also with strong host-specificity (Cotin-Galvan et al., 2016). At a worldwide scale, differences among *Frankia* communities are partly correlated with differences in EM communities (Põlme et al., 2014). From North America to Mexico, the similarities between EM communities also support a co-migration with their host (Kennedy et al., 2011). Together, these results suggest shared histories at broad scales, for the plant, *Frankia* and EM fungi.

If alder populations, *Frankia* and EM fungi communities have undergone a shared history, the bacterial and the fungal communities should have both survived in the Colchis, and alders should host more endemic symbionts in this refugium. Moreover, if South Caucasus has been a refugium and a recolonization source for Europe, European specific symbionts should be present in Georgia, together with endemic symbionts, especially in the Colchis. Therefore, the main objectives of the present study are to (1) document the diversity of the microbial symbionts communities of *A. glutinosa* subsp*. barbata* in Georgia and determine whether the Colchis floodplain hosts endemic symbionts, (2) test if EM fungi and *Frankia* communities are both more species rich in the Colchis, (3) examine how recent climatic history has imprinted the distribution of alder symbionts and their communities at a larger scale, by comparing our results to recent studies on the Hyrcanian forest.

## Material and Methods

### Sampling sites

**Figure 1:**
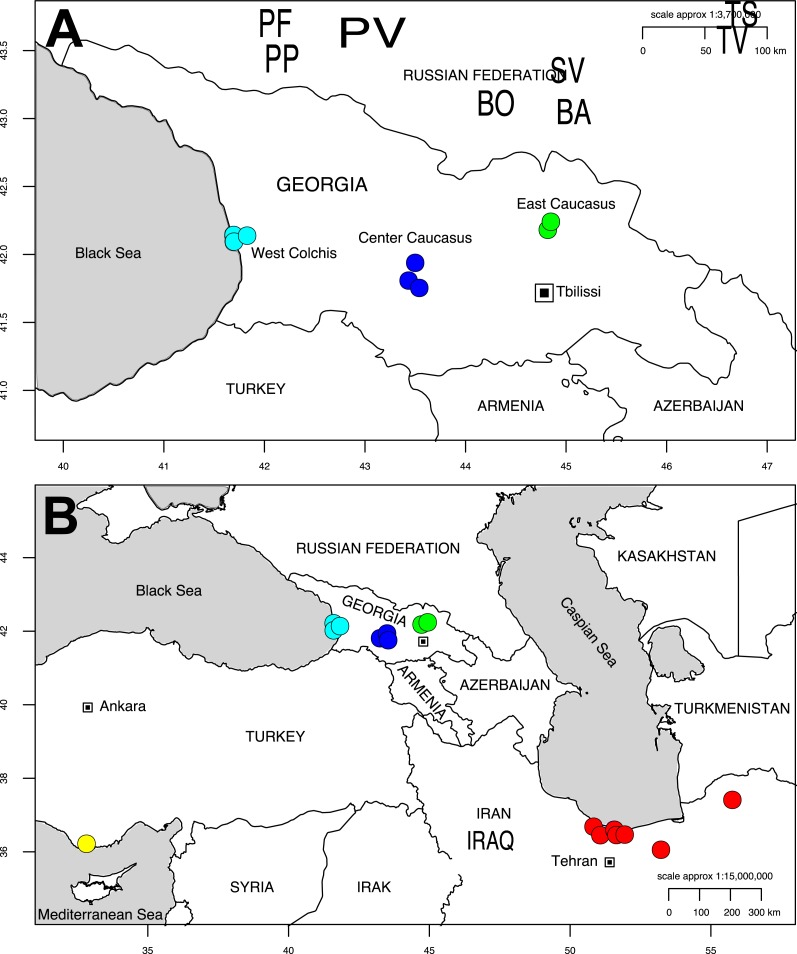
Map of Georgia (A) pointing out the sampling sites for *Alnus glutinosa* subsp. *barbata* in Western (Cyan), Central (Blue) and Eastern (Green) Georgia. Only Western sites belong to the Colchis floodplain. Map showing our study sites and sampling sites from previous studies on *Alnus subcordata* from the Hyrcanian forests in Iran in red, and *Alnus orientalis* from Turkey in Yellow (B). The two maps were produced with the R package OpenStreetMap.

Eight sites were studied in Georgia, including sites representing both large and isolated *Alnus* stands located from 0 to 1,800 m elevation. Three sites were located in the Colchis, considered as a refugium (PV, PP, PF). Five sites were located in the recolonized zone, three in central Georgia near the water separation line that splits Western and Eastern Georgia (BA, BO and SV), and two in Eastern Georgia at the Caucasus footstep (TS and TR; Fig. 1A, Table S1 of Appendix S1). Climatic data (19 variables) were retrieved from the Worldclim database (http://www.worldclim.org) through R raster package (Hijmans & Van Etten, 2012).

### Plant, ectomycorrhizal fungi and *Frankia* sampling

For each site, six trees were sampled, separated by at least 10 m as in Roy et al. (2013). One leaf per tree was dried in silica gel. Roots were collected after tracing roots up to one meter from the trunk and circa 50 cm of roots were collected per tree and kept in soil at 4°C until laboratory processing. In the laboratory, roots were washed under tap water over a 500 µm wide grid, and examined under a binocular microscope in distilled water. A minimum of 16 ectomycorrhizae per tree were picked, and separately kept in 2% CTAB buffer at 4°C for one week and then at −20°C. All *Frankia* nodules were stored in 2% CTAB buffer (100 mM Tris HCl pH8; 1.4 M NaCl; 20 mM Na_2_EDTA; 2% *N*-Acetyl-*NNN*-trimethyl ammonium bromide).

### *Alnus* phylogenetic position and genetic diversity

One cm^2^ of leaf was ground to extract DNA using the Wizard genomic DNA purification kit (Promega, Charbonnières les Bains, France) as in Rochet et al. (2011). The Internal Transcribed Spacer (ITS) of the plant nuclear ribosomal DNA was amplified using ITS1P (5′-TTATCATTTAGAGGAAGGAG-3′)—ITS4 primers (White et al., 1990) for each tree, following conditions of Rochet et al. (2011). The chloroplast gene *mat* K was amplified for one tree per population using the primer pairs Matk_1R_KIM and MatK_3F_KIM (Dunning & Savolainen, 2010) and sequenced following conditions of Rochet et al. (2011). Sequences were manually corrected, and deposited in Genbank under accession numbers KX897895 –KX897935 for ITS and KX897936 –KX897944 for *matK*). Reference sequences of European *Alnus* species were downloaded from Genbank Alignments were created with MUSCLE (Edgar, 2004). Phylogenies were computed using Raxml (Stamatakis, Hoover & Rougemont, 2008), by Maximum Likelihood analysis following a GTR model of evolution, and tested through a fast-bootstrap analysis (1,000 replicates) on the CIPRES website (Miller, Pfeiffer & Schwartz, 2010). Phylogenies allowed the placing of *A. glutinosa* subsp*. barbata* among other European alders and detect the occurrence of rare ITS variant in Georgia.

### Phylogenetic diversity of *Frankia* and comparison with European strains

The DNA was extracted from *Frankia* root nodules and three genes were amplified and sequenced: *dnaA*, *ftsZ* and *pgk*, with primers specific to *Frankia* as in Pozzi et al. (2015). Sequences were submitted to the EMBL (European Nucleotide Archive) under accession numbers LT616989 –LT617015 (*dnaA*), LT617016 –LT617048 (*ftsZ*), LT599862, LT599864, LT599865 and LT599870 –LT599890 (*pgk*). To test if *Frankia* sequences were phylogenetically more diverse in Georgia as compared to Europe, sequences from Pozzi et al. (2015), including strains isolated from *A. glutinosa* in Europe, were used as a reference. Alignments for each marker were built using MUSCLE and concatenated. A phylogeny was computed following the same methods as for the plant phylogeny.

All statistical analyses on community diversity were performed with R 2.3.4.4 (R Development Core Team, 2008). To describe the diversity of *Frankia* communities, pairwise Kimura’s 2-parameters distances were measured using ape (R) based on the *dnaA*, *ftsZ* and *pgk* alignment (concatenated). The histogram of pairwise distances measured from this alignment showed a distribution peak at 0.01 and a gap between 0.01 and 0.05 (absolute Kimura’s 2-parameters distances). Clusters of sequences more than 0.01 similar were considered as distinct molecular operational taxonomic units (MOTUs).

### Phylogenetic diversity of ectomycorrhizal fungi and comparison with European sequences

DNA extraction was performed on each EM root tip, using the Wizard genomic DNA purification kit (Promega, Charbonnières les Bains, France) as described in Rochet et al. (2011). The fungal ITS region was amplified using fungal universal primers ITS-1F/ITS-4 (Gardes & Bruns, 1993; White et al., 1990). PCR conditions were the same as in Rochet et al. (2011). Amplification products were sequenced by the MilleGen company (Labège, France). Sequences were manually corrected using 4Peaks 1.7.1 (Griekspoor & Groothuis, 2017) and deposited in Genbank (accession numbers KX897613 –KX897894).

Fungal sequences were compared to Genbank and UNITE http://unite.ut.ee/ (Kõljalg et al., 2005) databases using the BLAST algorithm (Altschul et al., 1990), which allowed the identification of fungal genera. To compare our sequences with MOTUs previously detected in Europe, all sequences produced recently from *Alnus* roots in Europe (Põlme et al., 2013; Roy et al., 2013) were downloaded. Sequences were aligned separately for each genus with MUSCLE (http://www.drive5.com/muscle/) (Edgar, 2004), and groups of sequences more than 97% similar were delineated with MOTHUR (Schloss et al., 2009), a threshold used in previous publications on *Alnus* and commonly accepted to delineate fungal MOTUs (Nilsson et al., 2008). Sequences that had between 93 and 97% similarity with Genbank or Unite sequences were identified from the BLAST result at the genus level only (e.g., “genus sp.”). Sequences that had no close similarity (>97%) with Genbank or Unite sequence were considered as putatively endemic to Georgia. For the specialist genus *Alnicola*, reference sequences produced from European specimens were downloaded from Genbank (Moreau, Peintner & Gardes, 2006; Rochet et al., 2011) and aligned with the sequences from the present study. Based on the alignment and phylogeny, computed using methods described above, *Alnicola* MOTUs were identified to the species level, and rare ITS variants were detected from the alignment.

### Diversity patterns of symbiont communities in Georgia

For *Frankia* and EM fungi, the species richness (alpha diversity) was measured at the site level, and the beta diversity was measured through Bray–Curtis distance, which takes species abundance into account. Species accumulation curves at the scale of Georgia and Chao 1 diversity indices were computed to reflect gamma diversity. The spatial autocorrelation of communities was tested by a multivariate autocorrelation test (multispati *r* test, ade4 package, Dray & Dufour, 2007). The correlation between beta diversity and distance was tested by a Mantel test (999 permutations). Because these tests were not statistically significant, no vector of spatial weights was integrated in the following tests. The difference in community structure (measured by the Bray– Curtis distance) between the three sampling zones was tested by a permutational multivariate analysis of variance (perMANOVA). Similarities between communities were visualized by a non-metric multidimensional scaling (NMDS). The correlation of NMDS structure with bioclimatic factors, latitude, longitude, and elevation was tested by permutations tests (envfit function, vegan package in R, Oksanen et al., 2007). The same analysis was computed for *Frankia* and for EM fungi together and separately.

**Figure 2:**
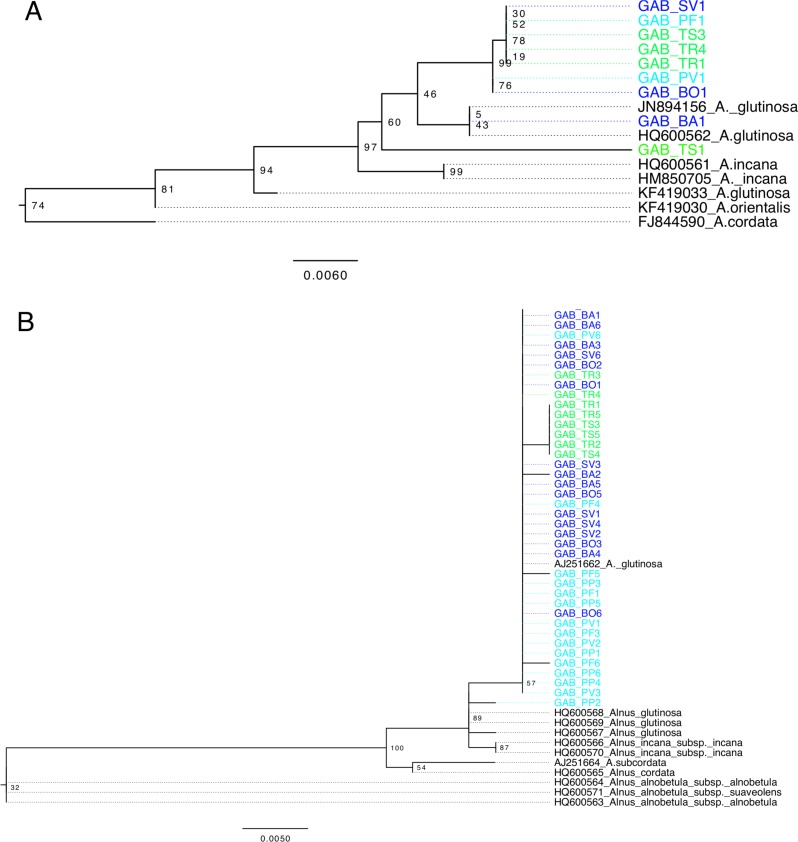
*Alnus* phylogeny, based on *matK* (A) and ITS (B), computed by maximum likelihood, following a GTR + I model of evolution, and tested by 1,000 bootstrap replicates. Sequences of *Alnus* isolated from Georgia are highlighted according to region as shown in Fig. 1. Sequences are named according to their sampling site.

### Comparison between EM fungi communities from Colchis and Hyrcan refugia

Only sequences of EM fungi were compared because our sequencing strategy for *Frankia* differs from Põlme et al. (2014). To compare EM communities between Colchis and the Hyrcanian forests, sequences produced by Põlme et al. (2013, sampling sites positioned on Fig. 1B) were downloaded from UNITE. Sampling strategy for EM root tips (5–6 trees sampled per site) and sequencing methods (Sanger sequencing of individual root tips, using the ITS marker) were sufficiently similar to be compared in a single analysis. Fungal sequences produced from mycorrhizae of *Alnus subcordata* CA Mey. and *A. glutinosa* in Iran, and *A. orientalis* Decne. in Turkey, were aligned with sequences from the present study, and MOTUs were delineated for each genus as previously described. A matrix of MOTUs x sites was built, including Turkish, Georgian and Iranian sites (see Table S2 of Appendix S1). We detected the occurrence of endemic or shared MOTU based on this matrix, We tested if Colchis communities were as species-rich as communities associated with *A. subcordata* in the Hyrcanian forests by analyzing alpha diversity variance (ANOVA). Finally, we tested how the geographical distance and the distinct hosts explained the differences in community structure observed in this region of Tertiary refugia (including sites in refugia and outside).

## Results

### *Alnus* phylogenetic position and genetic diversity at a regional scale

All *matK* sequences of *A. glutinosa* from Europe and *A. glutinosa* subsp*. barbata* clustered into a monophyletic clade (97% bootstrap, Fig. 2A) distinct from *A. incana* (L.) Moench. but monophyly of Georgian sequences was not supported. Two sequences from the Central and Eastern populations (TS1 and BA1) showed 11–27 nucleotide differences with other sequences from Georgia (Fig. 2A), and were more similar to sequences from Europe. The monophyletic clade of other Georgian sequences was characterized by eleven unique positions. Based on the ITS phylogeny, differences between *A. glutinosa* and *A. incana* were not supported, and only two nucleotides were variable between the two species (Fig. 2B). Besides a low bootstrap support, sequences from Georgia clustered in a monophyleticclade that also includes one European *A. glutinosa* sequence. The sequences from Eastern Georgia (all TS and part of TR) were characterized by one nucleotide difference with other sequences from Georgia.

### Phylogenetic diversity of *Frankia* and comparison with European strains

**Figure 3:**
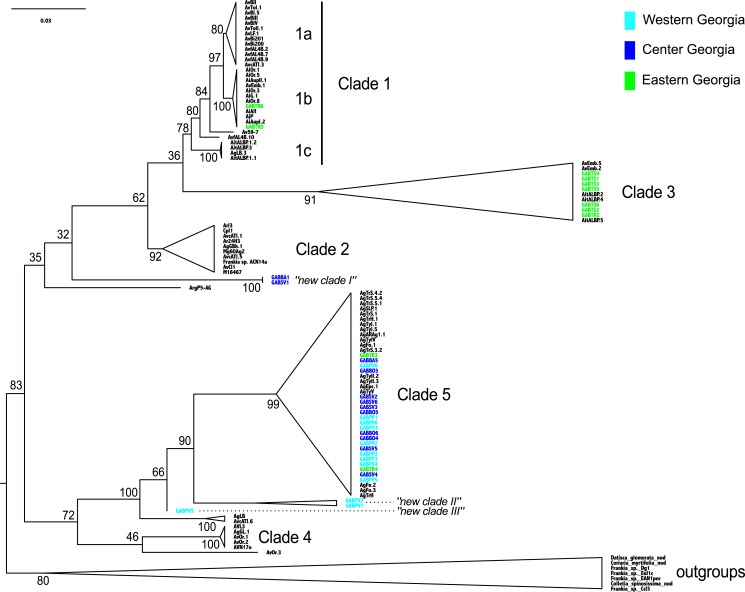
*Frankia* phylogeny, based on *dnaA*, *ftsZ*, and *pgk* genes, computed by maximum likelihood, following a GTR + I model of evolution, and tested by 1,000 bootstrap replicates. Sequences of *Frankia* isolated from Georgia are highlighted according to region as shown in Fig. 1. Sequences are named according to their sampling sites, and reference sequences are named as in Pozzi et al. (2015).

The *Frankia* strains detected in Georgia did not cluster into a monophyletic clade, and the twelve MOTUs were scattered throughout the phylogeny (clades 1b, 3 and 5 of Pozzi et al., 2015): clade 3 and 5 comprised three and four MOTUs respectively, and “new clade II” gathered two distinct MOTUs. All MOTUs but one belonged to clades commonly associated with *A. glutinosa* in Europe (Fig. 3). Two sequences from the Eastern population (TR) belonged to a monophyletic clade, frequently associated with *A. alnobetula* (Erhr.) K Koch (clade 1b, Fig. 3). Three sequences from Western populations were not similar to any other known sequence (new clade II and III, Fig. 3). Finally, two Central Georgia sequences clustered in an isolated lineage (new clade I, Fig. 3).

### Phylogenetic diversity of ectomycorrhizal fungi and comparison with European sequences

DNA was extracted and PCR-amplified from a total of 732 individual root tips: 484 of them produced ITS sequences among which 328 met our quality threshold. The four Helotiales and non-EM fungi sequences were not taken into account in our analysis (one Xylariaceae, four *Rhizoctonia*, one Diaporthales). Twenty-nine MOTUs were recorded among which 21 belonged to Basidiomycota and 8 to Ascomycota (91.2% and 8.8% of ectomycorrhizae, respectively). Seven *Alnicola* species were detected, all strictly identical to species found in Europe. Of interest, two new ITS variants of *Alnicola xanthophylla* were detected in Central Georgia*,* differing by five positions from their European relatives. Among non-*Alnicola* EM fungi, five new MOTUs were detected in Georgia. Three were Ascomycota (*Tuber* sp.*, Tarzetta* sp.*, Peziza* sp.) and two Basidiomycota (*Inocybe* spp.). These five possibly endemic EM fungi were found in Central and Eastern Georgia.

**Figure 4:**
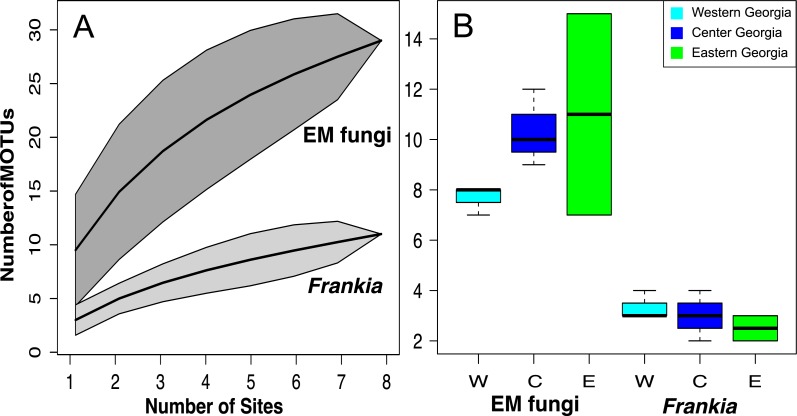
MOTU accumulation curves computed for all Georgian site for *Frankia* and EM fungi (A) and species richness per site in West, Central and East Georgia for *Frankia* and EM fungi (B). Shaded area represent 95% confidence intervals.

### Diversity patterns of symbiont communities in Georgia

**Figure 5:**
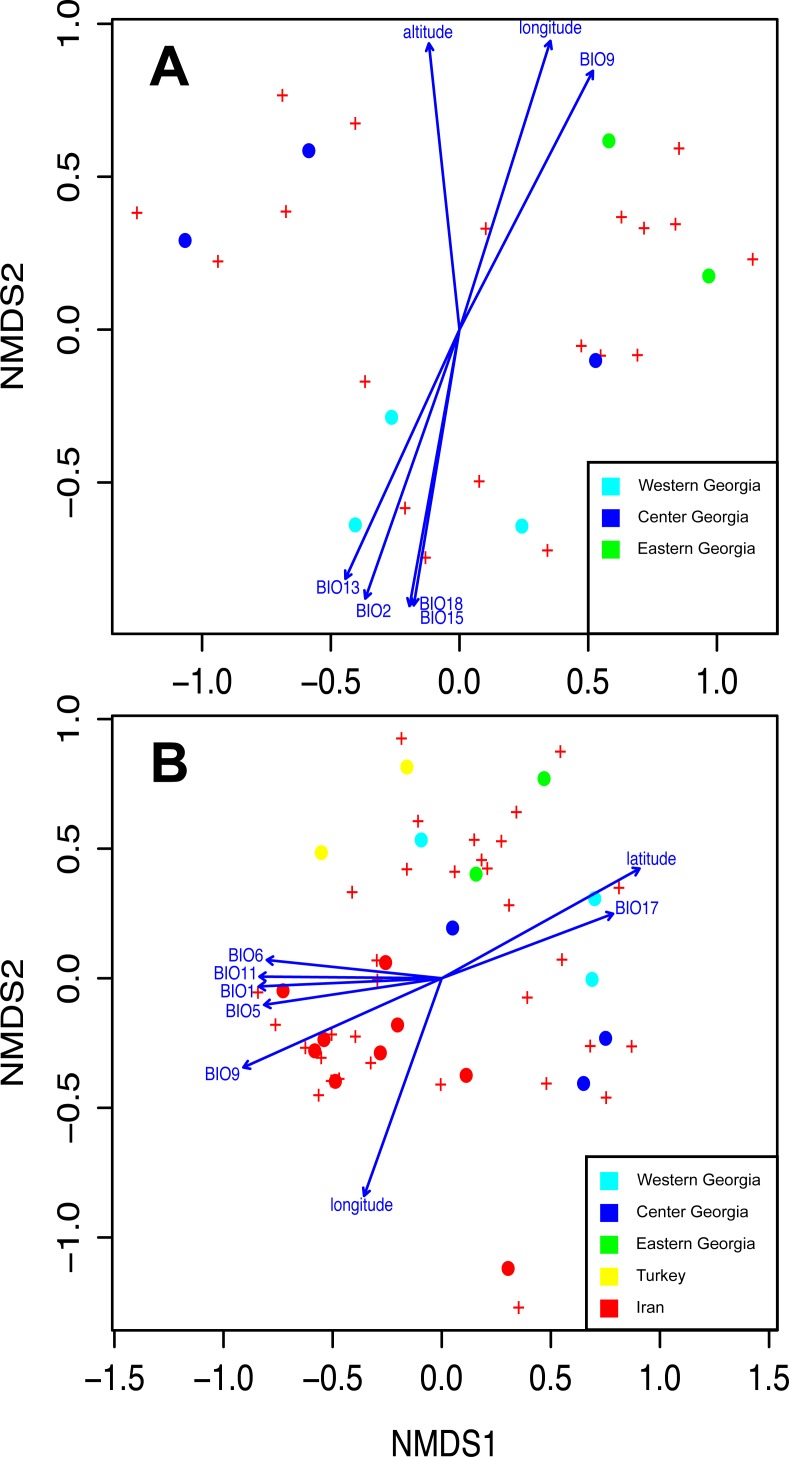
Non-Metric Multidimensional Scaling (NMDS) ordering the differences between EM and *Frankia* communities and showing the correlation with environmental and geographical parameters in Georgia (A) and at a wider scale between Georgia, Iran and Turkey for EM fungi only (B). Significant variables are represented (*P* > 0.05 according to environmental fitting tests; see Table S3 of Appendix S1). BIO1, temperature seasonality; BIO2, maximum temperature of the warmest month; BIO5, precipitation of the warmest month; BIO6, precipitation of the driest month; BIO9, precipitation of the warmest quarter; BIO11, isothermality; BIO13, precipitation of the wettest quarter; BIO15, annual mean temperature; BIO17, mean temperature of the driest quarter and BIO18, precipitation of the coldest quarter.

Species accumulation curves show that sampling was not saturated, especially for EM fungi. According to Chao 1 estimates, up to 26.7 *Frankia* and 44.7 EM MOTUs could occur in Georgia (Fig. 4A). Species richness per site varied between 7 and 15 MOTUs for fungi, and from 2 to 4 for *Frankia* (Fig. 4B). Compared to the recolonized zone, *Frankia* and EM communities were neither significantly richer (Kruskal-Wallis test, *p* = 0.48 and *p* = 0.17 respectively, Fig. 4B) nor different based on community composition (perMANOVA on Bray–Curtis distances, *p* = 0.40 and *p* = 0.19 for *Frankia* and EM fungi, respectively). Communities were not spatially autocorrelated (multivariate spatial autocorrelation test, *p*-value = 0.338 for EM fungi, *p* = 0.348 for *Frankia*), and the spatial distance did not correlate with Bray–Curtis distances at least for EM fungi (Mantel test, *p* = 0.16). For *Frankia*, the beta diversity was marginally correlated with distance (Mantel test, *p* = 0.0594). For the two types of symbionts, Bray–Curtis distances between sites were significantly correlated with longitude and several bioclimatic factors linked with precipitations (see Table S3 of Appendix S1, Fig. 5A).

### Comparison between EM communities from Colchis and Hyrcanian refugia

The dominant MOTUs in Georgia were rare in Turkey and Iran, and *vice versa* (see Table S2 of Appendix S1). Among the 68 MOTUs, three were shared between Georgia and adjacent countries (*Paxillus adelphus* and two MOTUs belonging to the genus *Tomentella*), nine between Georgia and Iran (MOTUs belonging to the genera *Alnicola*, *Cortinarius*, *Inocybe*, *Sebacina*, and *Tarzetta*), and none between Georgia and Turkey. Interestingly, the genus *Inocybe* and several Ascomycota were detected on alder roots both in Iran and in Georgia. At the community level, the Georgian EM communities were not more diverse than those on other alder species in Iran or Turkey (ANOVA, *p*-value = 0.14). The spatial auto-correlation test was still not significant (multivariate autocorrelation test, *p* = 0.072). Variation in beta-diversity was strongly correlated with geographic distances (Mantel test, *r* = 0.51, *p* < 0.0001) and was also related to host species (42% of variation explained, PERMANOVA test, *p* = 0.001). Finally, longitude, latitude, altitude, and bioclimatic variables related to temperature all were significantly correlated with beta-diversity (Fig. 5B, Table S3 of Appendix S1).

## Discussion

Our first objective was to document the diversity of symbiont communities associated with *Alnus glutinosa* ssp. *barbata* in Georgia, especially in the Colchis floodplain, a glacial and Tertiary refugium for alders. In all, 29 EM MOTUs and 12 *Frankia* MOTUs were uncovered, and Chao 1 estimates showed that twice as many species may occur there. For *Frankia*, the common European lineages were detected, together with five new lineages, which is an exceptional pattern considering the extensive worldwide *Frankia* database (Pozzi et al., 2015). Georgia proved to be a region of high *Frankia* diversity, probably of ancient origin as suggested by the long branches supporting Caucasian lineages (Fig. 2). For EM fungi, most Georgian MOTUs (24 out of 29) were already reported from Europe, on roots of *A. glutinosa*, *A. incana* and *A. cordata* (Loisel.) Duby. No endemic MOTU was detected for the most specialized lineages, but two unique ITS variants of *Alnicola xanthophylla* were found in Central Georgia. From previous study on the genus *Alnicola* (Moreau, Peintner & Gardes, 2006; Rochet et al., 2011), the ITS is known to be often not variable within species, and 1.1% genetic variability is exceptional for this genus. Although a deeper sampling may reveal more fungi, these results reveal that endemism is relatively rare in the Caucasus region for EM fungi, even the more specialized ones associated with alders. Interestingly, a similar analysis on corticioid fungi in South Caucasus has also pointed out the low number of endemics, and the high similarity with European communities (Ghobad-Nejhad et al., 2012).

The presence of five endemic *Frankia*, but no endemic specialized EM fungi was not expected but could be explained by differences in dispersal and survival abilities between the two types of symbionts. A free-living stage (outside the host) has been reported for the *Frankia* strains with low host specificity (Maunuksela et al., 1999), but aside from this case, most uncultured *Frankia* are highly dependent of their host (Pozzi et al., 2015). As with *Frankia*, most EM fungi cannot survive in the soil without their host (Lindahl & Tunlid, 2015). *Frankia* produces spores belowground and is dispersed by water or animals (Chaia, Wall & Huss-Danell, 2010) and sometimes by wind (Dawson, 2007) leading to short-distance dispersal, and perhaps greater endemism whereas *Alnicola* are wind-dispersed and potentially disperse over large distances. Among fungi, two potential endemic species belong to a wind-dispersed genus (*Inocybe).* Although species of *Inocybe* are regularly detected on alder roots (Bogar & Kennedy, 2013; Roy et al., 2013; Põlme et al., 2013), current knowledge on this genus is still too limited to determine if these *Inocybe* sp. are specific to *Alnus* or if their occurrence reflects a local adaptation to a particular habitat. More generally, Ryberg et al. (2008) have shown that the ITS evolves faster in the genus *Inocybe*, and that environmental sequencing often reveals new species in this genus. Among the EM fungi lineages that form hypogeous (below-ground) fruitbodies, three potential endemic Ascomycota species were detected. The degree of specificity of these species towards *Alnus* is still unknown, and two genera, *Tuber* and *Tarzetta*, are often described as pioneer species with broad host ranges. The presence of endemics for these two genera might be due to their long-term persistence in refugia. Similarly, the persistence of the black truffle *Tuber melanosporum* Vittad. during glaciations has been shown in Spanish (García-Cunchillos et al., 2014) and Italian refugia (Rubini et al., 2005).

Our second objective was to assess whether the symbiont communities and their host populations follow a shared history at the scale of Georgia. We hypothesized that sites in the refugium would host more endemic and diverse communities of both *Frankia* and EM fungi, but we did not confirm our hypothesis. First, the host population were genetically more diverse in the recolonized zone, where several unique ITS and *matK* variants were detected for *A. glutinosa subsp. barbata*. These variants were clearly distinct from other sequences of European *A. glutinosa* and this result confirms that Georgia, and not only the Colchis, is a hot spot of genetic diversity for the *A. glutinosa* complex (pointed out by King & Ferris, 1998). Interestingly, all endemic EM MOTUs were detected in Central and Eastern sites, associated with the most genetically diverse host populations. However, the species richness of *Frankia* communities showed a different distribution, as three endemic strains were detected in the Colchis, and two in the Central population. These different distributions of genetic diversity and species richness suggest that all symbionts do not necessarily survive with their host in refugia. Moreover, the occurrence of unique ITS and *matK* variants for alders and endemic EM fungi in Central and Eastern sites show that isolated host populations could also act as refugia, especially in mountain ranges, as highlighted by recent reviews on the Mediterranean basin (Médail & Diadema, 2009; Dobrowski, 2011; Keppel et al., 2012). The differences between the Colchis and Central and Eastern populations could reflect different biogeographic histories, as shown for snails and amphibians (Tarkhnishvili, 1996; Tarkhnishvili, Thorpe & Arntzen, 2000; Pokryszko et al., 2011) but also correlate with changes in annual precipitation and summer temperatures, as pinpointed by our analyses (Fig. 5).

Our third objective was to compare the Colchis and the Hyrcanian communities, associated with two alder species, *A. glutinosa* subsp. *barbata* and *A. subcordata*, that are both considered to be Tertiary relics. The two refugia hosted distinct EM communities, and differences were strongly explained by both host identity and geographic distance. The lack of shared MOTUs, even abundant ones (Table S2 of Appendix S1), explains these differences, and suggests a limited spore flow between the two regions. The scarcity of alder stands between these two regions in the steppe landscapes could indeed limit dispersal, and maintain isolation between the two regions, as observed for plants (Nakhutsrishvili et al., 2015) and corticioid fungi (Ghobad-Nejhad et al., 2012). Interestingly, both *A. subcordata* and *A. glutinosa subsp. barbata* associate with several *Inocybe,* Ascomycota and especially *Tuber*. Additional Tertiary refugia should be investigated in Asia to determine if these associations are also relic from the Tertiary, or derive from local and independent adaptations in relatively dry regions for alders. Indeed, *Tuber* and hypogeous fungi are known to be particularly resistant to drought (Richard et al., 2011; Herzog et al., 2013 ) and abundant in dry habitats (Zambonelli et al., 2014).

For microbes, biogeographic studies are relatively recent (Martiny et al., 2006), partly because the “everything is everywhere” idiom was long said to be the rule. Now, Beringia has been pinpointed as a hotspot for high-latitude fungi (Geml et al., 2010; Bellemain et al., 2013), and could have been a glacial refugium for the fungi. In Europe, the Perigord truffle *Tuber melanosporum*, also an EM species, has more genetically diverse populations in southern-Italy (Rubini et al., 2005) and Spain (García-Cunchillos et al., 2014). Finally, the anther smut fungus *Microbotryum* sp., an obligate parasite of Caryophyllaceae, has apparently followed its host during recent glaciation (Vercken et al., 2010). Here, we focus on entire communities and show on the contrary, that alpha diversity was not higher in the refugium than in the recolonized zone. The results from analyzing communities versus populations are not necessarily coupled (Vellend & Geber, 2005), but theoretically area size and immigration have parallel effects on species richness and genetic diversity (Vellend & Geber, 2005). The Theory of Island Biogeography (MacArthur & Wilson, 1967) predicts that smaller refugia can favor local extinctions (Kadmon & Allouche, 2007), and moreover, bottleneck effects may also reduce local diversity (Bennett & Provan, 2008). On the other hand, recent experiments on alders have shown that their specificity might be controlled by pH and nitrogen availability (Huggins et al., 2014), which may reduce the influence of host biogeography on symbiont community diversity. In this framework, it is even more puzzling to observe more endemic EM fungi and rare plant ITS variant in the isolated populations from Central and Eastern Georgia. As the endemic *Inocybe* and *Tuber* are not usually associated with alders, we suspect that these taxa have only recently colonized alder roots, a host shift probably favored by the actual isolation of alders in this region.

## Conclusions

Wiens & Donoghue (2004) have already highlighted the chasm between historical biogeography and community ecology, and suggested that more links should be drawn between these topics. In a second step, studies on symbiont populations may help confirming the lack of isolation in refugia. The recent studies of alder refugia in Europe could be used as a framework to study more in depth alder symbiont biogeography (Douda et al., 2014; Mandák et al., 2016a; Mandák et al., 2016b). Finally, the occurrence of endemic OTUs detected for both EM fungi and *Frankia* outside of refugia highlight their relevance to biodiversity conservation not only in Tertiary and glacial refugia, but also in isolated host populations.

##  Supplemental Information

10.7717/peerj.3479/supp-1 Supplemental Information 1 Appendix S1 Additional Supporting Information may be found in the online version of this article: Appendix S1. Supplementary Tables. Table S1. Site characteristics, location, and occurrence of *Frankia* and EM MOTUs among sites. *putative endemic MOTUs Table S2. Matrix reporting occurrence of EM MOTU among sites located in Turkey, Iran and Georgia. Table S3. Correlation of climatic factors and geographic position with NMDS structure for all symbionts in Georgia, *Frankia* only, EM fungi only, and for EM fungi in Georgia, Turkey and Iran.

10.7717/peerj.3479/supp-2 Supplemental Information 1 ITS sequences for fungi

10.7717/peerj.3479/supp-3 Supplemental Information 2 Plant ITS from Georgia

10.7717/peerj.3479/supp-4 Supplemental Information 3 matK sequence from Georgia

## Additional Information and Declarations

### Field Study Permissions

The following information was supplied relating to field study approvals (i.e., approving body and any reference numbers): Studies were handled thanks to Professor George Nakhutsrishvili from Tbilisi Botany institute. No permits were required outside of parks in Georgia.

### DNA Deposition

The following information was supplied regarding the deposition of DNA sequences: Plant sequences are deposited at Genbank (KX897895 –KX897935 for ITS and KX897936 –KX897944 for matK). *Frankia* Sequences were submitted to the EMBL (European Nucleotide Archive) under accession numbers LT616989 –LT617015 (dnaA), LT617016 –LT617048 (ftsZ), LT599862, LT599864, LT599865 and LT599870 –LT599890 (pgk). Fungal sequences are deposited at Genbank under accession numbers KX897613 –KX897894.

### Data Availability

The following information was supplied regarding data availability: The sequences are deposited at GenBank and EMBL, and raw data is included in Appendix S1.
